# A Novel Low-Cost Simulation Model for Point-of-Care Ultrasound Intussusception Practice

**DOI:** 10.7759/cureus.61016

**Published:** 2024-05-24

**Authors:** Jasmine Williams, Jessica Doctor, Kristine Jeffers, Melissa Myers

**Affiliations:** 1 Emergency Medicine, Brooke Army Medical Center, Fort Sam Houston, USA; 2 Emergency Medicine, San Antonio Military Medical Center, San Antonio, USA

**Keywords:** simulation trainer, emergency medicine training, emergency medicine, pediatric ultrasound, ultrasound

## Abstract

Pediatric intussusception is a relatively common yet serious condition where prompt diagnosis is crucial. Point-of-care ultrasound (POCUS) has proven accurate for diagnosing this disease and can expedite both diagnosis and treatment. Previous research has shown that emergency physicians can diagnose intussusception with acceptable sensitivity and specificity but require prior training in recognizing the pathology. Despite the disease's relative frequency, any individual physician rarely encounters it, making a simulation model vital for learning this ultrasound modality. We created a model using low-cost, easily available components that can be used to train emergency physicians to diagnose intussusception on POCUS.

## Introduction

Ileocecal intussusception is a common surgical abdominal emergency in pediatric patients with a reported incidence of 33 cases per 100,000 life births in the first year of life [1]. Peak age for presentation is five to ten months old and ranges up to three years. One of the most common presentations is an infant presenting with colicky abdominal pain, irritability, vomiting, and bloody stools [2]. The condition is often misdiagnosed initially because it mimics other more benign conditions such as viral gastroenteritis. The delay in diagnosis can lead to complications such as obstruction, ischemia, necrosis, and perforation [3].

Ultrasound is the preferred modality in assessment for intussusception due to its lack of ionizing radiation and ability to obtain detailed visualization of the digestive system in pediatric patients [4]. Classical findings of intussusception on ultrasound are based on the orientation of the probe and include the “target sign” caught in the transverse view or the “pseudokidney sign” caught in the longitudinal view [4]. When performed by experts, ultrasound has close to 100% sensitivity and specificity for diagnosing intussusception [4]. Many centers, however, do not have 24-hour access to experts who can perform ultrasounds. This has raised significant interest in the role of point-of-care ultrasound (POCUS) in the diagnosis of intussusception.

A recent systematic review and meta-analysis showed that overall POCUS is 95% specific and 99% sensitive for the diagnosis [5]. Pediatric Emergency Physicians, despite higher exposure to pediatric pathology, may struggle with the use of POCUS for intussusception. Specialists in pediatric emergency medicine (PEM) face barriers as well. 65% of PEM physicians felt they did not have enough time to learn POCUS and 76.7% identified personal discomfort with their POCUS skills [6]. This likely also stems from the lack of competency felt by PEM fellows still in training, with less than half of fellows feeling competent in identifying intussusception despite their program including it in their curriculum [7]. In another study looking at the rate of misdiagnosis of intussusception, a brief POCUS training led to lower rates of misdiagnosis in both junior and senior physicians [3].

Because of the importance of the pathology and the difficulty in learning to identify this on ultrasound without practice, we designed a low-cost do-it-yourself (DIY) model to simulate intussusception. By using the model, emergency medicine residents can learn to recognize intussusception on ultrasound, which they can then incorporate into their clinical practice.

## Technical report

We created a low-cost model using easily obtained materials to meet our goal of creating a widely accessible and affordable model. The model was created using silicone tubing (3/8 inch outer diameter x 1/4 inch inner diameter x 10 feet) from a home improvement store, and soybean oil, psyllium fiber supplement, gelatin, food dye, and a baking pan were purchased from a grocery store (Table 1).

**Table 1:** Cost summary

Material	Cost
Silicone tubing	$2.40 per foot
Soybean oil	$4
Baking dish (3 quart)	$7
Gelatin (32 0.25 oz packets)	$11
Psyllium fiber supplement	$14
Food coloring	$4
Cloth pins	$2
Total cost	$66

**Figure 1:**
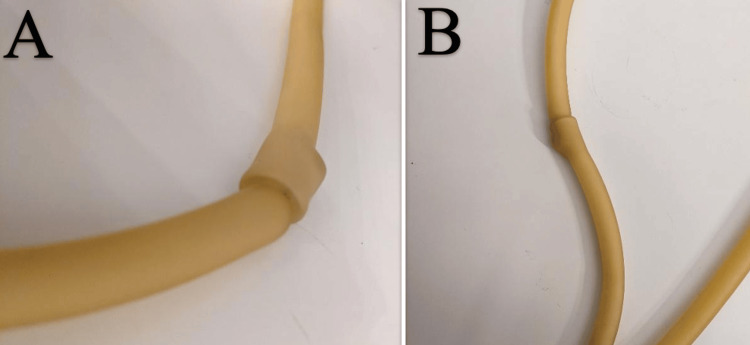
A) Long view of the tubing. B) Second long view of the tubing from a different angle

The silicone tubing was cut into several sections to create multiple models. In each section, a portion of the tubing was folded into itself to simulate intussusception pathology (Figure 1).

Next, vegetable oil was poured into the tube to eliminate air and allow ultrasound waves to penetrate without air artifacts. Both ends of the tube were then tied into a knot to prevent leaking of the vegetable oil.

**Figure 2:**
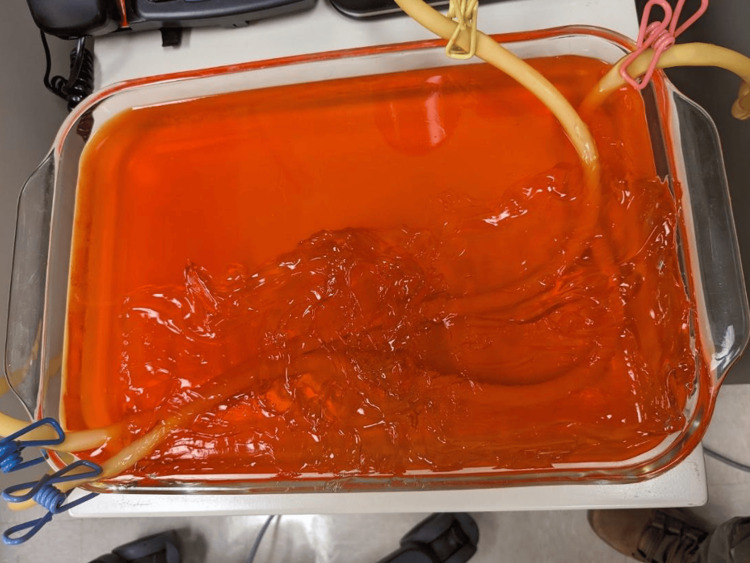
Second iteration intussusception model

The tubes were then placed in a three-quart baking dish and scattered randomly to simulate bowel and set with gelatin mixed with a psyllium fiber supplement in a 1:1 ratio [8]. Dark food coloring was mixed with the gelatin to make visualization of the simulated intussusception more difficult. Clothespins were used to keep the tubing from floating to the top of the gelatin mixture while setting. The final model consisted of the gelatin mixture with oil-filled tubing in a baking dish (Figure 2).

**Figure 3:**
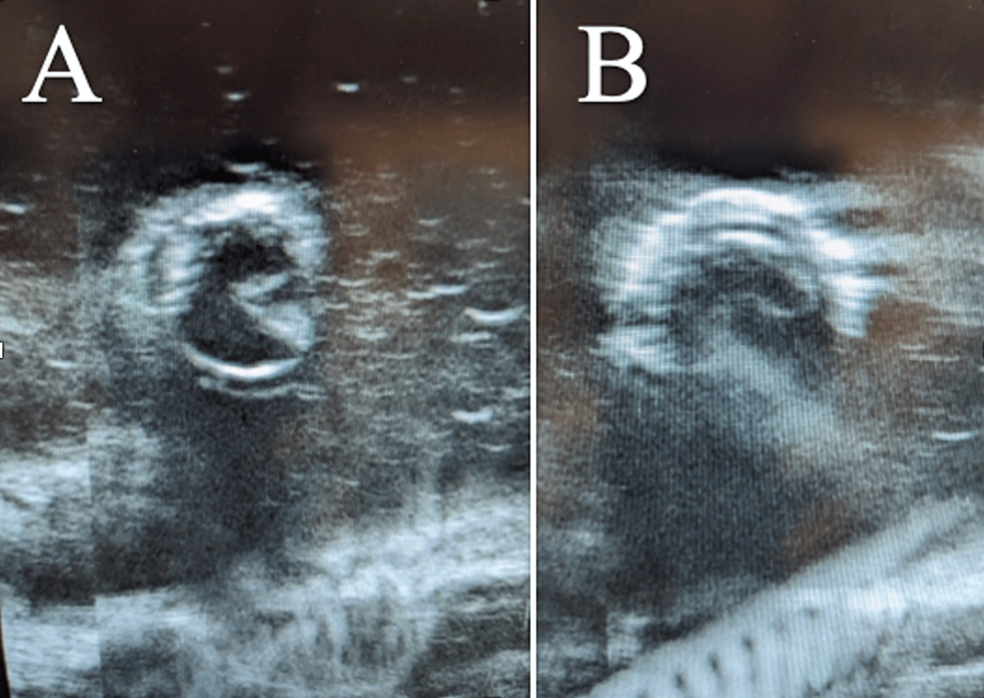
A) Transverse view of the intussusception model visualized by ultrasound. B) Second view of the model at a different point

The model successfully simulated intussusception pathology (Figure 3). This model was used by 19 residents during four training sessions over a period of two weeks.

## Discussion

**Figure 4:**
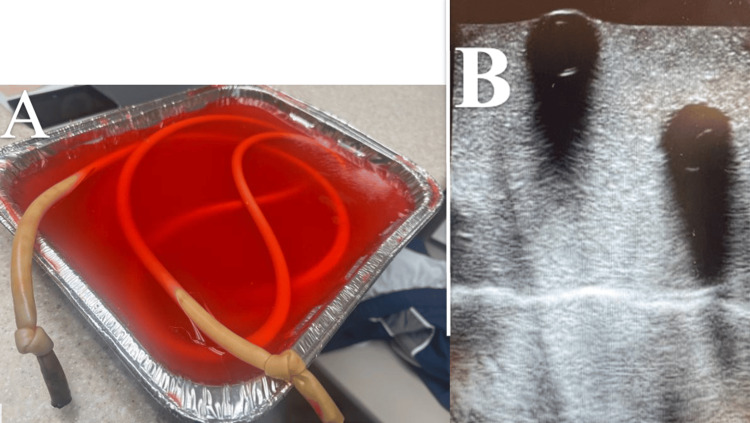
A) The completed initial model. B) Shadowing that prevented visualization of the simulated intussusception

Our model was successful in the basic task of simulating intussusception; however, we noted several areas for improvement in future iterations. No commercial models are currently available, and this ultrasound model is taught in a primarily didactic format. An initial model was constructed with tubing that caused significant shadowing and prevented visualization of the intussusception simulation (Figure 4). There were two likely causes for the initial shadowing. First, we used carbonated water to mimic bowel gas. which caused air to become trapped within the lumen of the model. Second, we had difficulty invaginating the tubing. A second model was constructed using vegetable oil, which resolved both problems. We suggest that oil be used when constructing future models. Noticeably, the longitudinal view of the model only showed the top layer of the tubing, which we suspect was due to the ultrasound waves' inability to penetrate through either the thick double layer of silicone or air trapped between the two layers in which oil was unable to fill.

The gelatin model was relatively fragile. We suggest that cellulose instead of psyllium fiber could be used to thicken the gelatin mixture, which would allow the model to last longer without spoiling. An increased amount of the thickening agent could also be utilized to make the model more opaque as well as increase its integrity as some learners were heavy-handed and pushed through the gelatin medium. Increasing the concentration of food coloring may have also helped the gelatin mixture be more opaque. 

For future models, areas for experimentation could include using a smaller baking dish to fill more completely with tubing and better simulate pediatric bowel. We did not attempt to compare multiple types of oil, such as sunflower or canola oil, and it is possible that a different type of oil would have produced a higher fidelity model. Future research could include a direct comparison of models using differing types of oil. In addition, we recommend constructing multiple models with some having simulated normal bowel to increase the realism of the simulation environment.

## Conclusions

Intussusception is a relatively common surgical emergency in pediatric patients, which is best diagnosed on ultrasound. In many small or rural emergency departments, ultrasound technicians are unavailable much of the time so relying on radiology to perform ultrasound can significantly increase the time to diagnosis and potentially harm the patient. POCUS can improve care for these patients by decreasing the time to diagnosis and definitive treatment but requires training on the part of the user. This introduces a problem in training, as the pathology is relatively common but infrequently seen by any individual physician. In this article, we describe the creation of a low-cost model using materials that can be easily found at a grocery and home goods store. The use of a low-cost model similar to the one described in this technical report can improve the training and readiness of emergency medicine physicians to diagnose this disease at the bedside with POCUS.
